# Tetra­kis[μ-1,4-bis­(4,5-dihydro-1,3-oxazol-2-yl)benzene-κ^2^ *N*:*N*′]tetra­kis­(μ-methano­lato-κ^2^ *O*:*O*)bis­(μ-perchlorato-κ^2^ *O*:*O*′)tetra­copper(II) bis­(perchlorate)

**DOI:** 10.1107/S160053681102705X

**Published:** 2011-07-13

**Authors:** Chun-Wei Yeh, Fu-Chang Huang, Ay Jong, Maw-Cherng Suen

**Affiliations:** a Department of Chemistry, Chung-Yuan Christian University, Chung-Li 32023, Taiwan; b Department of Civil and Environmental Engineering, Nanya Institute of Technology, Chung-Li 32091, Taiwan; c Department of Chemical and Material Engineering, Nanya Institute of Technology, Chung-Li 32091, Taiwan; d Department of Material and Fiber, Nanya Institute of Technology, Chung-Li 32091, Taiwan

## Abstract

The title tetra­nuclear Cu^II^ complex, [Cu_4_(C_12_H_12_N_2_O_2_)_4_(CH_3_O)_4_(ClO_4_)_2_](ClO_4_)_2_, is located around an inversion center. Each Cu^II^ atom is coordinated by two *cis*-O atoms from two bridging methano­late anions and two *cis*-N atoms from two bridging 1,4-bis­(4,5-dihydro-1,3-oxazol-2-yl)benzene (*L*) ligands in the basal plane, and is further coordinated by one O atom of the bridging perchlorate anion, forming a distorted square-pyramidal geometry. The Cu⋯Cu separations in the recta­ngular core are 2.9878 (11) and 6.974 (1) Å. In the asymmetric unit, there are two *L* ligands with a *syn* conformation. In one *L* ligand, the dihedral angles between the central benzene ring and the terminal 4,5-dihydro-1,3-oxazol-2-yl mean planes are 22.1 (4) and 33.1 (4)°, and in the other *L* ligand the corresponding dihedral angles are 29.3 (4) and 29.9 (4)°. The uncoordinated perchlorate anion is linked with the complex mol­ecules *via* weak C—H⋯O hydrogen bonds.

## Related literature

For related structures, see: Wang *et al.* (2008 ▶, 2011 ▶).
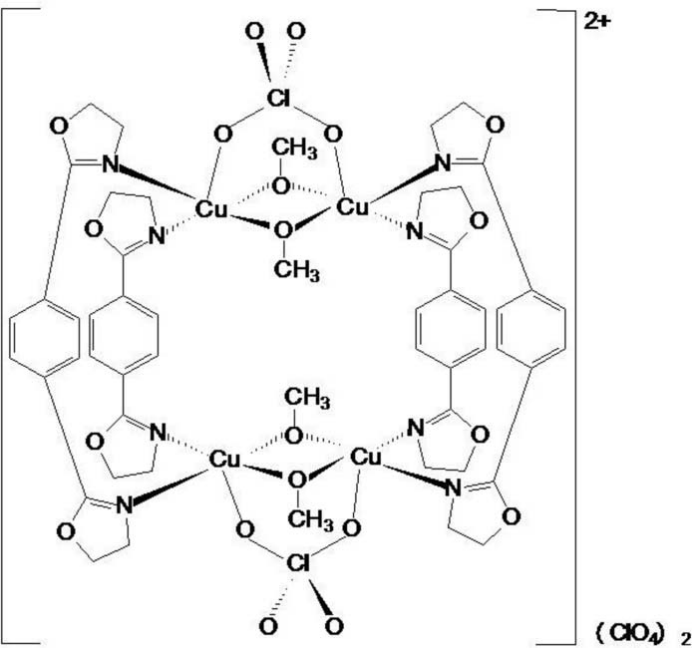


## Experimental

### 

#### Crystal data

[Cu_4_(C_12_H_12_N_2_O_2_)_4_(CH_3_O)_4_(ClO_4_)_2_](ClO_4_)_2_
*M* *_r_* = 1641.04
Monoclinic, 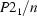

*a* = 8.3508 (8) Å
*b* = 16.9820 (18) Å
*c* = 22.337 (2) Å
β = 93.936 (2)°
*V* = 3160.1 (6) Å^3^
*Z* = 2
Mo *K*α radiation
μ = 1.59 mm^−1^
*T* = 297 K
0.30 × 0.15 × 0.07 mm

#### Data collection

Bruker APEXII CCD diffractometer
Absorption correction: multi-scan (*SADABS*; Bruker, 2000 ▶) *T*_min_ = 0.647, *T*_max_ = 0.897
17717 measured reflections
6219 independent reflections
3428 reflections with *I* > 2σ(*I*)
*R*_int_ = 0.077

#### Refinement

*R*[*F* ^2^ > 2σ(*F* ^2^)] = 0.058
*wR*(*F* ^2^) = 0.186
*S* = 1.00
6219 reflections
433 parameters
H-atom parameters constrained
Δρ_max_ = 1.65 e Å^−3^
Δρ_min_ = −1.30 e Å^−3^

### 

Data collection: *APEX2* (Bruker, 2010 ▶); cell refinement: *SAINT* (Bruker, 2010 ▶); data reduction: *SAINT*; program(s) used to solve structure: *SHELXTL* (Sheldrick, 2008 ▶); program(s) used to refine structure: *SHELXTL*; molecular graphics: *DIAMOND* (Brandenburg, 2010 ▶); software used to prepare material for publication: *SHELXTL*.

## Supplementary Material

Crystal structure: contains datablock(s) I, global. DOI: 10.1107/S160053681102705X/xu5263sup1.cif

Structure factors: contains datablock(s) I. DOI: 10.1107/S160053681102705X/xu5263Isup2.hkl

Additional supplementary materials:  crystallographic information; 3D view; checkCIF report

## Figures and Tables

**Table 1:** Hydrogen-bond geometry (Å, °)

*D*—H⋯*A*	*D*—H	H⋯*A*	*D*⋯*A*	*D*—H⋯*A*
C2—H2*A*⋯O13^i^	0.97	2.55	3.281 (11)	132
C3—H3*B*⋯O11^i^	0.97	2.42	3.228 (11)	141
C8—H8*A*⋯O5^ii^	0.93	2.60	3.476 (8)	157
C9—H9*A*⋯O6	0.93	2.57	3.465 (8)	161
C12—H12*A*⋯O13^iii^	0.97	2.54	3.450 (12)	157
C24—H24*A*⋯O2^iv^	0.97	2.54	3.270 (9)	132
C26—H26*B*⋯O9	0.96	2.36	3.240 (14)	152

Symmetry codes: (i) 
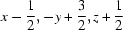
; (ii) 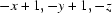; (iii) 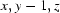; (iv) 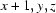.

**Fig. 1.**
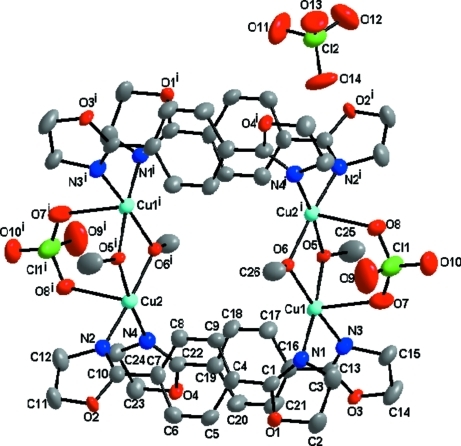
The molecule structure showing the coordination environment of the Cu(II) atoms. Thermal ellipsoids are drawn at 30% probability level, and H atoms are omitted for clarity. Symmetry code: (i) -x + 1, -y + 1, -z.

## supplementary crystallographic information

### Comment

Several Ag(I) and Cu(II) coordination polymers containing 1,4-bis(4,5-dihydro-1,3-oxazol-2-yl)benzene ligands has been reported, which show various two-dimensional networks (Wang, *et al.*, 2008; Wang, *et al.*, 2011). In the title molecule, [Cu_4_(C_12_H_12_N_2_O_2_)_4_(CH_3_O)_4_(ClO_4_)_2_](ClO_4_)_2_, the tetranuclear Cu(II) ion are located around an inversion center. The Cu(II) atom is bounded by two *cis-*O atoms from two bridging meyhoxide anions and two *cis-*N atoms from two *L* ligands in the basal plane and the Cu(II) atom is also bonded one oxygen atom of the bridging perchlorate anion forming a highly distorted square-pyramidal geometry. The Cu···Cu separation in the rectangular core are 2.9878 (11) and 6.974 (1) Å, while those are separated by the bridging perchlorate anions and *L* ligands. In the present work, the structure of the *L* ligand has been determined to explore its ligand conformation. In the crystal structure of the title compound the molecule is in a *syn* conformation. This conformation is different from those in the Ag(I) and Cu(II) complexes, which is *anti* (Wang, *et al.*, 2008).

### Experimental

1,4-Bis(4,5-dihydro-1,3-oxazol-2-yl)benzene (1.0 mmol) was placed in a flask containing 20 ml methanol and copper perchlorate (1.0 mmol) was added. The mixture was then refluxed for 24 h to afford a blue solution with some blue solid. The solution was filtered and the blue crystals were obtained by slow diffusion of diethyl ether into the filtrate of the compound for several weeks. These were washed with methanol and diethylether and collected in 75.0% yield.

### Refinement

H atoms were constrained to ideal geometries, with C—H = 0.93 (phenyl), 0.96 (methyl) or 0.97 (methylene) Å and *U*_iso_(H) = 1.2*U*_eq_(C).

### Crystal data

**Table:**

[Cu_4_(C_12_H_12_N_2_O_2_)_4_(CH_3_O)_4_(ClO_4_)_2_](ClO_4_)_2_	*F*(000) = 1672
*M**_r_* = 1641.04	*D*_x_ = 1.725 Mg m^−^^3^
Monoclinic, *P*2_1_/*n*	Mo *K*α radiation, λ = 0.71073 Å
Hall symbol: -P 2yn	Cell parameters from 3303 reflections
*a* = 8.3508 (8) Å	θ = 2.2–23.9°
*b* = 16.9820 (18) Å	µ = 1.59 mm^−^^1^
*c* = 22.337 (2) Å	*T* = 297 K
β = 93.936 (2)°	Parallelepiped, blue
*V* = 3160.1 (6) Å^3^	0.30 × 0.15 × 0.07 mm
*Z* = 2	

### Data collection

**Table:**

Bruker APEXII CCD diffractometer	6219 independent reflections
Radiation source: fine-focus sealed tube	3428 reflections with *I* > 2σ(*I*)
graphite	*R*_int_ = 0.077
φ and ω scans	θ_max_ = 26.0°, θ_min_ = 1.5°
Absorption correction: multi-scan (*SADABS*; Bruker, 2000)	*h* = −10→8
*T*_min_ = 0.647, *T*_max_ = 0.897	*k* = −13→20
17717 measured reflections	*l* = −27→27

### Refinement

**Table:**

Refinement on *F*^2^	Primary atom site location: structure-invariant direct methods
Least-squares matrix: full	Secondary atom site location: difference Fourier map
*R*[*F*^2^ > 2σ(*F*^2^)] = 0.058	Hydrogen site location: inferred from neighbouring sites
*wR*(*F*^2^) = 0.186	H-atom parameters constrained
*S* = 1.00	*w* = 1/[σ^2^(*F*_o_^2^) + (0.0854*P*)^2^ + 5.5939*P*] where *P* = (*F*_o_^2^ + 2*F*_c_^2^)/3
6219 reflections	(Δ/σ)_max_ < 0.001
433 parameters	Δρ_max_ = 1.65 e Å^−^^3^
0 restraints	Δρ_min_ = −1.30 e Å^−^^3^

### Special details

**Table:**

Experimental. Refinement of *F*^2^ against ALL reflections. The weighted *R*-factor *wR* and goodness of fit *S* are based on *F*^2^, conventional *R*-factors *R* are based on *F*, with *F* set to zero for negative *F*^2^. The threshold expression of *F*^2^ > σ(*F*^2^) is used only for calculating *R*-factors(gt) *etc*. and is not relevant to the choice of reflections for refinement. *R*-factors based on *F*^2^ are statistically about twice as large as those based on *F*, and *R*- factors based on ALL data will be even larger.
Geometry. All e.s.d.'s (except the e.s.d. in the dihedral angle between two l.s. planes) are estimated using the full covariance matrix. The cell e.s.d.'s are taken into account individually in the estimation of e.s.d.'s in distances, angles and torsion angles; correlations between e.s.d.'s in cell parameters are only used when they are defined by crystal symmetry. An approximate (isotropic) treatment of cell e.s.d.'s is used for estimating e.s.d.'s involving l.s. planes.
Refinement. Refinement of *F*^2^ against ALL reflections. The weighted *R*-factor *wR* and goodness of fit *S* are based on *F*^2^, conventional *R*-factors *R* are based on *F*, with *F* set to zero for negative *F*^2^. The threshold expression of *F*^2^ > σ(*F*^2^) is used only for calculating *R*-factors(gt) *etc*. and is not relevant to the choice of reflections for refinement. *R*-factors based on *F*^2^ are statistically about twice as large as those based on *F*, and *R*- factors based on ALL data will be even larger.

### Fractional atomic coordinates and isotropic or equivalent isotropic displacement parameters (Å^2^)

**Table:**

	*x*	*y*	*z*	*U*_iso_*/*U*_eq_	
C1	0.1122 (8)	0.4667 (4)	0.1591 (3)	0.0359 (16)	
C2	−0.0067 (9)	0.5004 (5)	0.2428 (3)	0.050 (2)	
H2A	0.0396	0.4747	0.2787	0.060*	
H2B	−0.1127	0.5199	0.2503	0.060*	
C3	0.0998 (9)	0.5658 (5)	0.2232 (3)	0.047 (2)	
H3A	0.0367	0.6111	0.2095	0.057*	
H3B	0.1756	0.5819	0.2557	0.057*	
C4	0.1474 (8)	0.4084 (4)	0.1125 (3)	0.0342 (16)	
C5	0.1018 (9)	0.3300 (5)	0.1204 (3)	0.0448 (19)	
H5A	0.0513	0.3155	0.1546	0.054*	
C6	0.1313 (9)	0.2745 (4)	0.0779 (3)	0.0415 (18)	
H6A	0.1002	0.2225	0.0831	0.050*	
C7	0.2083 (8)	0.2962 (4)	0.0266 (3)	0.0344 (16)	
C8	0.2554 (8)	0.3736 (4)	0.0189 (3)	0.0400 (17)	
H8A	0.3082	0.3877	−0.0149	0.048*	
C9	0.2240 (8)	0.4301 (4)	0.0612 (3)	0.0376 (17)	
H9A	0.2537	0.4822	0.0556	0.045*	
C10	0.2276 (8)	0.2353 (4)	−0.0197 (3)	0.0349 (16)	
C11	0.1477 (10)	0.1274 (5)	−0.0718 (4)	0.053 (2)	
H11A	0.0627	0.1262	−0.1036	0.063*	
H11B	0.1642	0.0745	−0.0560	0.063*	
C12	0.3012 (9)	0.1601 (5)	−0.0943 (3)	0.0471 (19)	
H12A	0.3867	0.1214	−0.0903	0.057*	
H12B	0.2852	0.1756	−0.1361	0.057*	
C13	0.5597 (8)	0.4671 (5)	0.2289 (3)	0.0330 (16)	
C14	0.5841 (11)	0.5144 (5)	0.3226 (3)	0.057 (2)	
H14A	0.6812	0.5300	0.3459	0.069*	
H14B	0.5022	0.5019	0.3499	0.069*	
C15	0.5281 (10)	0.5786 (5)	0.2801 (3)	0.0470 (19)	
H15A	0.4280	0.6014	0.2914	0.056*	
H15B	0.6080	0.6199	0.2786	0.056*	
C16	0.5799 (8)	0.4058 (4)	0.1829 (3)	0.0372 (17)	
C17	0.6125 (8)	0.4276 (4)	0.1250 (3)	0.0375 (17)	
H17A	0.6150	0.4806	0.1144	0.045*	
C18	0.6415 (8)	0.3699 (4)	0.0829 (3)	0.0387 (17)	
H18A	0.6594	0.3844	0.0438	0.046*	
C19	0.6439 (8)	0.2904 (4)	0.0988 (3)	0.0364 (16)	
C20	0.6098 (11)	0.2702 (5)	0.1575 (3)	0.055 (2)	
H20A	0.6066	0.2174	0.1684	0.066*	
C21	0.5813 (10)	0.3267 (5)	0.1987 (3)	0.052 (2)	
H21A	0.5626	0.3122	0.2378	0.063*	
C22	0.6849 (8)	0.2287 (4)	0.0567 (3)	0.0345 (16)	
C23	0.7830 (10)	0.1081 (5)	0.0355 (3)	0.0455 (19)	
H23A	0.7213	0.0607	0.0414	0.055*	
H23B	0.8955	0.0943	0.0347	0.055*	
C24	0.7231 (9)	0.1506 (4)	−0.0216 (3)	0.0427 (18)	
H24A	0.8100	0.1592	−0.0476	0.051*	
H24B	0.6392	0.1206	−0.0434	0.051*	
C25	0.7168 (9)	0.6445 (6)	0.1504 (4)	0.065 (3)	
H25A	0.7898	0.6699	0.1252	0.098*	
H25B	0.7607	0.5948	0.1638	0.098*	
H25C	0.7007	0.6773	0.1845	0.098*	
C26	0.1317 (8)	0.6419 (5)	0.0591 (3)	0.049 (2)	
H26A	0.1182	0.6662	0.0202	0.074*	
H26B	0.0872	0.6754	0.0883	0.074*	
H26C	0.0775	0.5920	0.0581	0.074*	
N1	0.1845 (7)	0.5304 (4)	0.1729 (2)	0.0360 (14)	
N2	0.3399 (7)	0.2294 (3)	−0.0559 (2)	0.0341 (13)	
N3	0.5057 (7)	0.5363 (4)	0.2212 (2)	0.0382 (14)	
N4	0.6591 (6)	0.2277 (3)	−0.0004 (2)	0.0347 (13)	
O1	−0.0137 (6)	0.4464 (3)	0.1911 (2)	0.0457 (13)	
O2	0.1089 (6)	0.1818 (3)	−0.0245 (2)	0.0445 (13)	
O3	0.6144 (6)	0.4476 (3)	0.28487 (19)	0.0437 (13)	
O4	0.7583 (6)	0.1666 (3)	0.0827 (2)	0.0468 (13)	
O5	0.5680 (5)	0.6320 (3)	0.11751 (19)	0.0355 (11)	
O6	0.2978 (5)	0.6303 (3)	0.07467 (19)	0.0334 (11)	
O7	0.3217 (10)	0.7040 (4)	0.2147 (3)	0.093 (2)	
O8	0.3809 (8)	0.7958 (4)	0.1440 (3)	0.0692 (18)	
O9	0.1149 (11)	0.7691 (7)	0.1666 (5)	0.146 (4)	
O10	0.2738 (10)	0.8399 (5)	0.2321 (3)	0.104 (3)	
O11	0.6921 (15)	0.8936 (6)	−0.1375 (4)	0.156 (4)	
O12	0.7923 (10)	0.9813 (6)	−0.0703 (5)	0.134 (4)	
O13	0.5292 (11)	0.9929 (5)	−0.1101 (4)	0.114 (3)	
O14	0.6030 (9)	0.8924 (6)	−0.0449 (4)	0.120 (3)	
Cl1	0.2917 (4)	0.77931 (15)	0.19370 (10)	0.0846 (9)	
Cl2	0.6500 (3)	0.93970 (13)	−0.09112 (9)	0.0592 (6)	
Cu1	0.38371 (9)	0.58253 (5)	0.14926 (3)	0.0313 (2)	
Cu2	0.53626 (9)	0.29352 (5)	−0.06120 (3)	0.0312 (2)	

### Atomic displacement parameters (Å^2^)

**Table:**

	*U*^11^	*U*^22^	*U*^33^	*U*^12^	*U*^13^	*U*^23^
C1	0.039 (4)	0.044 (4)	0.024 (3)	−0.001 (4)	0.003 (3)	0.007 (3)
C2	0.054 (5)	0.067 (6)	0.033 (4)	−0.010 (4)	0.021 (4)	−0.011 (4)
C3	0.046 (4)	0.055 (5)	0.043 (4)	−0.007 (4)	0.020 (4)	−0.014 (4)
C4	0.032 (4)	0.044 (4)	0.026 (3)	−0.002 (3)	0.001 (3)	−0.001 (3)
C5	0.061 (5)	0.048 (5)	0.027 (4)	−0.007 (4)	0.013 (3)	0.005 (3)
C6	0.048 (4)	0.037 (4)	0.041 (4)	−0.008 (4)	0.008 (3)	0.002 (3)
C7	0.034 (4)	0.038 (4)	0.031 (4)	−0.003 (3)	−0.003 (3)	−0.002 (3)
C8	0.045 (4)	0.039 (4)	0.037 (4)	−0.004 (4)	0.010 (3)	−0.001 (3)
C9	0.044 (4)	0.038 (4)	0.032 (4)	−0.009 (3)	0.007 (3)	0.005 (3)
C10	0.040 (4)	0.034 (4)	0.030 (4)	−0.002 (3)	−0.001 (3)	0.004 (3)
C11	0.065 (5)	0.044 (5)	0.051 (5)	−0.019 (4)	0.011 (4)	−0.018 (4)
C12	0.054 (5)	0.038 (4)	0.049 (5)	−0.002 (4)	0.003 (4)	−0.014 (4)
C13	0.032 (4)	0.048 (5)	0.019 (3)	−0.005 (3)	0.002 (3)	0.005 (3)
C14	0.076 (6)	0.070 (6)	0.025 (4)	0.022 (5)	0.002 (4)	0.000 (4)
C15	0.063 (5)	0.055 (5)	0.023 (4)	0.004 (4)	0.004 (3)	0.001 (3)
C16	0.038 (4)	0.048 (5)	0.025 (3)	0.002 (3)	−0.001 (3)	0.003 (3)
C17	0.046 (4)	0.032 (4)	0.035 (4)	0.008 (3)	0.006 (3)	0.006 (3)
C18	0.048 (4)	0.044 (4)	0.025 (3)	0.005 (4)	−0.001 (3)	0.006 (3)
C19	0.037 (4)	0.041 (4)	0.031 (4)	0.003 (3)	−0.001 (3)	0.006 (3)
C20	0.090 (6)	0.038 (5)	0.038 (4)	0.001 (5)	0.019 (4)	0.006 (4)
C21	0.078 (6)	0.051 (5)	0.030 (4)	0.002 (5)	0.013 (4)	0.008 (4)
C22	0.040 (4)	0.034 (4)	0.030 (4)	0.002 (3)	0.004 (3)	0.008 (3)
C23	0.053 (5)	0.042 (5)	0.041 (4)	0.010 (4)	0.000 (4)	0.001 (4)
C24	0.059 (5)	0.036 (4)	0.034 (4)	0.007 (4)	0.009 (4)	0.002 (3)
C25	0.046 (5)	0.090 (7)	0.058 (5)	−0.008 (5)	−0.010 (4)	0.024 (5)
C26	0.038 (4)	0.061 (5)	0.048 (5)	0.001 (4)	−0.003 (3)	0.019 (4)
N1	0.041 (3)	0.040 (4)	0.028 (3)	−0.003 (3)	0.006 (3)	0.001 (3)
N2	0.040 (3)	0.029 (3)	0.033 (3)	−0.001 (3)	0.004 (3)	0.000 (3)
N3	0.047 (4)	0.039 (4)	0.028 (3)	0.004 (3)	0.005 (3)	0.003 (3)
N4	0.035 (3)	0.036 (3)	0.034 (3)	0.000 (3)	0.007 (3)	0.001 (3)
O1	0.042 (3)	0.057 (3)	0.040 (3)	−0.012 (3)	0.018 (2)	−0.007 (3)
O2	0.046 (3)	0.038 (3)	0.050 (3)	−0.015 (2)	0.013 (2)	−0.014 (2)
O3	0.056 (3)	0.049 (3)	0.026 (2)	0.014 (3)	0.000 (2)	0.008 (2)
O4	0.064 (3)	0.044 (3)	0.032 (3)	0.014 (3)	−0.004 (2)	0.005 (2)
O5	0.031 (2)	0.045 (3)	0.029 (2)	0.002 (2)	−0.0024 (19)	0.012 (2)
O6	0.030 (2)	0.038 (3)	0.031 (2)	0.004 (2)	−0.0010 (19)	0.005 (2)
O7	0.121 (6)	0.075 (5)	0.084 (5)	0.013 (5)	0.025 (4)	0.034 (4)
O8	0.092 (5)	0.065 (4)	0.053 (4)	0.001 (4)	0.028 (3)	0.006 (3)
O9	0.090 (6)	0.188 (11)	0.156 (9)	0.002 (7)	−0.034 (6)	−0.040 (8)
O10	0.137 (7)	0.095 (6)	0.085 (5)	−0.013 (5)	0.041 (5)	−0.040 (5)
O11	0.247 (12)	0.142 (9)	0.081 (6)	0.053 (9)	0.029 (7)	−0.032 (6)
O12	0.100 (6)	0.104 (7)	0.202 (10)	−0.027 (6)	0.030 (6)	0.003 (7)
O13	0.131 (7)	0.100 (6)	0.111 (6)	0.038 (5)	0.004 (5)	0.049 (5)
O14	0.085 (5)	0.179 (9)	0.098 (6)	0.014 (6)	0.023 (4)	0.091 (6)
Cl1	0.163 (3)	0.0498 (14)	0.0452 (13)	−0.0013 (16)	0.0409 (16)	−0.0001 (11)
Cl2	0.0819 (16)	0.0525 (13)	0.0454 (11)	0.0083 (12)	0.0204 (11)	0.0094 (10)
Cu1	0.0342 (5)	0.0370 (5)	0.0227 (4)	0.0011 (4)	0.0028 (3)	0.0038 (4)
Cu2	0.0350 (5)	0.0335 (5)	0.0251 (4)	0.0001 (4)	0.0030 (3)	0.0037 (4)

### Geometric parameters (Å, °)

**Table:**

C1—N1	1.268 (9)	C18—C19	1.394 (10)
C1—O1	1.355 (8)	C18—H18A	0.9300
C1—C4	1.480 (10)	C19—C20	1.404 (9)
C2—O1	1.472 (8)	C19—C22	1.463 (10)
C2—C3	1.507 (10)	C20—C21	1.361 (11)
C2—H2A	0.9700	C20—H20A	0.9300
C2—H2B	0.9700	C21—H21A	0.9300
C3—N1	1.493 (8)	C22—N4	1.280 (8)
C3—H3A	0.9700	C22—O4	1.334 (8)
C3—H3B	0.9700	C23—O4	1.473 (9)
C4—C9	1.399 (9)	C23—C24	1.522 (10)
C4—C5	1.399 (10)	C23—H23A	0.9700
C5—C6	1.373 (10)	C23—H23B	0.9700
C5—H5A	0.9300	C24—N4	1.503 (9)
C6—C7	1.399 (9)	C24—H24A	0.9700
C6—H6A	0.9300	C24—H24B	0.9700
C7—C8	1.388 (10)	C25—O5	1.415 (8)
C7—C10	1.479 (9)	C25—H25A	0.9600
C8—C9	1.385 (10)	C25—H25B	0.9600
C8—H8A	0.9300	C25—H25C	0.9600
C9—H9A	0.9300	C26—O6	1.420 (8)
C10—N2	1.283 (8)	C26—H26A	0.9600
C10—O2	1.343 (8)	C26—H26B	0.9600
C11—O2	1.456 (8)	C26—H26C	0.9600
C11—C12	1.515 (10)	N1—Cu1	1.987 (6)
C11—H11A	0.9700	N2—Cu2	1.979 (6)
C11—H11B	0.9700	N3—Cu1	2.003 (6)
C12—N2	1.478 (9)	N4—Cu2	1.988 (6)
C12—H12A	0.9700	O5—Cu1	1.931 (4)
C12—H12B	0.9700	O5—Cu2^i^	1.947 (4)
C13—N3	1.266 (9)	O6—Cu2^i^	1.935 (5)
C13—O3	1.343 (7)	O6—Cu1	1.945 (4)
C13—C16	1.482 (10)	O7—Cl1	1.380 (7)
C14—O3	1.446 (9)	O8—Cl1	1.407 (6)
C14—C15	1.500 (10)	O9—Cl1	1.566 (9)
C14—H14A	0.9700	O10—Cl1	1.353 (7)
C14—H14B	0.9700	O11—Cl2	1.364 (8)
C15—N3	1.499 (9)	O12—Cl2	1.433 (9)
C15—H15A	0.9700	O13—Cl2	1.398 (8)
C15—H15B	0.9700	O14—Cl2	1.385 (7)
C16—C21	1.389 (10)	Cu1—Cu2^i^	2.9878 (11)
C16—C17	1.390 (9)	Cu2—O6^i^	1.935 (5)
C17—C18	1.391 (10)	Cu2—O5^i^	1.947 (4)
C17—H17A	0.9300	Cu2—Cu1^i^	2.9878 (11)
			
N1—C1—O1	117.5 (6)	N4—C22—O4	117.7 (6)
N1—C1—C4	128.9 (6)	N4—C22—C19	128.2 (6)
O1—C1—C4	113.6 (6)	O4—C22—C19	114.1 (6)
O1—C2—C3	102.9 (5)	O4—C23—C24	103.1 (6)
O1—C2—H2A	111.2	O4—C23—H23A	111.1
C3—C2—H2A	111.2	C24—C23—H23A	111.1
O1—C2—H2B	111.2	O4—C23—H23B	111.1
C3—C2—H2B	111.2	C24—C23—H23B	111.1
H2A—C2—H2B	109.1	H23A—C23—H23B	109.1
N1—C3—C2	104.0 (6)	N4—C24—C23	104.6 (5)
N1—C3—H3A	111.0	N4—C24—H24A	110.8
C2—C3—H3A	111.0	C23—C24—H24A	110.8
N1—C3—H3B	111.0	N4—C24—H24B	110.8
C2—C3—H3B	111.0	C23—C24—H24B	110.8
H3A—C3—H3B	109.0	H24A—C24—H24B	108.9
C9—C4—C5	119.8 (6)	O5—C25—H25A	109.5
C9—C4—C1	121.6 (6)	O5—C25—H25B	109.5
C5—C4—C1	118.6 (6)	H25A—C25—H25B	109.5
C6—C5—C4	120.2 (6)	O5—C25—H25C	109.5
C6—C5—H5A	119.9	H25A—C25—H25C	109.5
C4—C5—H5A	119.9	H25B—C25—H25C	109.5
C5—C6—C7	120.0 (7)	O6—C26—H26A	109.5
C5—C6—H6A	120.0	O6—C26—H26B	109.5
C7—C6—H6A	120.0	H26A—C26—H26B	109.5
C8—C7—C6	120.1 (7)	O6—C26—H26C	109.5
C8—C7—C10	121.9 (6)	H26A—C26—H26C	109.5
C6—C7—C10	117.8 (6)	H26B—C26—H26C	109.5
C9—C8—C7	120.2 (6)	C1—N1—C3	106.5 (6)
C9—C8—H8A	119.9	C1—N1—Cu1	135.1 (5)
C7—C8—H8A	119.9	C3—N1—Cu1	118.0 (4)
C8—C9—C4	119.7 (7)	C10—N2—C12	106.7 (6)
C8—C9—H9A	120.1	C10—N2—Cu2	129.7 (5)
C4—C9—H9A	120.1	C12—N2—Cu2	123.5 (4)
N2—C10—O2	117.6 (6)	C13—N3—C15	107.7 (6)
N2—C10—C7	128.0 (6)	C13—N3—Cu1	129.0 (5)
O2—C10—C7	114.4 (6)	C15—N3—Cu1	122.8 (5)
O2—C11—C12	104.0 (6)	C22—N4—C24	106.7 (6)
O2—C11—H11A	111.0	C22—N4—Cu2	135.8 (5)
C12—C11—H11A	111.0	C24—N4—Cu2	116.9 (4)
O2—C11—H11B	111.0	C1—O1—C2	105.4 (5)
C12—C11—H11B	111.0	C10—O2—C11	106.7 (5)
H11A—C11—H11B	109.0	C13—O3—C14	106.7 (5)
N2—C12—C11	104.8 (6)	C22—O4—C23	107.7 (5)
N2—C12—H12A	110.8	C25—O5—Cu1	124.6 (4)
C11—C12—H12A	110.8	C25—O5—Cu2^i^	125.2 (5)
N2—C12—H12B	110.8	Cu1—O5—Cu2^i^	100.81 (19)
C11—C12—H12B	110.8	C26—O6—Cu2^i^	124.5 (4)
H12A—C12—H12B	108.9	C26—O6—Cu1	124.4 (4)
N3—C13—O3	116.8 (6)	Cu2^i^—O6—Cu1	100.7 (2)
N3—C13—C16	127.9 (6)	O10—Cl1—O7	120.9 (5)
O3—C13—C16	115.1 (6)	O10—Cl1—O8	115.8 (5)
O3—C14—C15	105.1 (6)	O7—Cl1—O8	111.1 (4)
O3—C14—H14A	110.7	O10—Cl1—O9	100.7 (6)
C15—C14—H14A	110.7	O7—Cl1—O9	100.0 (6)
O3—C14—H14B	110.7	O8—Cl1—O9	104.7 (5)
C15—C14—H14B	110.7	O11—Cl2—O14	109.5 (7)
H14A—C14—H14B	108.8	O11—Cl2—O13	111.1 (6)
N3—C15—C14	102.8 (6)	O14—Cl2—O13	111.4 (5)
N3—C15—H15A	111.2	O11—Cl2—O12	106.2 (7)
C14—C15—H15A	111.2	O14—Cl2—O12	108.3 (6)
N3—C15—H15B	111.2	O13—Cl2—O12	110.2 (6)
C14—C15—H15B	111.2	O5—Cu1—O6	76.15 (18)
H15A—C15—H15B	109.1	O5—Cu1—N1	173.9 (2)
C21—C16—C17	119.8 (7)	O6—Cu1—N1	98.4 (2)
C21—C16—C13	120.2 (6)	O5—Cu1—N3	95.2 (2)
C17—C16—C13	119.8 (6)	O6—Cu1—N3	171.1 (2)
C16—C17—C18	119.6 (7)	N1—Cu1—N3	90.1 (2)
C16—C17—H17A	120.2	O5—Cu1—Cu2^i^	39.79 (13)
C18—C17—H17A	120.2	O6—Cu1—Cu2^i^	39.52 (13)
C17—C18—C19	120.7 (6)	N1—Cu1—Cu2^i^	135.87 (17)
C17—C18—H18A	119.6	N3—Cu1—Cu2^i^	132.83 (17)
C19—C18—H18A	119.6	O6^i^—Cu2—O5^i^	76.02 (18)
C18—C19—C20	118.3 (7)	O6^i^—Cu2—N2	169.4 (2)
C18—C19—C22	122.0 (6)	O5^i^—Cu2—N2	93.5 (2)
C20—C19—C22	119.7 (6)	O6^i^—Cu2—N4	98.1 (2)
C21—C20—C19	121.0 (7)	O5^i^—Cu2—N4	173.4 (2)
C21—C20—H20A	119.5	N2—Cu2—N4	92.3 (2)
C19—C20—H20A	119.5	O6^i^—Cu2—Cu1^i^	39.77 (12)
C20—C21—C16	120.5 (7)	O5^i^—Cu2—Cu1^i^	39.40 (13)
C20—C21—H21A	119.7	N2—Cu2—Cu1^i^	130.64 (16)
C16—C21—H21A	119.7	N4—Cu2—Cu1^i^	135.84 (16)

Symmetry codes: (i) −*x*+1, −*y*+1, −*z*.

### Hydrogen-bond geometry (Å, °)

**Table:**

*D*—H···*A*	*D*—H	H···*A*	*D*···*A*	*D*—H···*A*
C2—H2A···O13^ii^	0.97	2.55	3.281 (11)	132
C3—H3B···O11^ii^	0.97	2.42	3.228 (11)	141
C8—H8A···O5^i^	0.93	2.60	3.476 (8)	157
C9—H9A···O6	0.93	2.57	3.465 (8)	161
C12—H12A···O13^iii^	0.97	2.54	3.450 (12)	157
C24—H24A···O2^iv^	0.97	2.54	3.270 (9)	132
C26—H26B···O9	0.96	2.36	3.240 (14)	152

Symmetry codes: (ii) *x*−1/2, −*y*+3/2, *z*+1/2; (i) −*x*+1, −*y*+1, −*z*; (iii) *x*, *y*−1, *z*; (iv) *x*+1, *y*, *z*.

## References

[bb1] Brandenburg, K. (2010). *DIAMOND* Crystal Impact GbR, Bonn, Germany.

[bb2] Bruker (2000). *SADABS* Bruker AXS Inc., Madison, Wisconsin, USA.

[bb3] Bruker (2010). *APEX2* and *SAINT* Bruker AXS Inc., Madison, Wisconsin, USA.

[bb4] Sheldrick, G. M. (2008). *Acta Cryst.* A**64**, 112–122. 10.1107/S0108767307043930 18156677

[bb5] Wang, Y.-H., Lee, H.-T. & Suen, M.-C. (2008). *Polyhedron*, **27**, 1177–1184.

[bb6] Wang, P.-N., Yeh, C.-W., Tsai, H.-A., Wang, J.-C. & Suen, M.-C. (2011). *Acta Cryst.* E**67**, m881. 10.1107/S1600536811020605 PMC3152049 21836874

